# Preparation of EGCG decorated, injectable extracellular vesicles for cartilage repair in rat arthritis

**DOI:** 10.1093/rb/rbab067

**Published:** 2021-11-18

**Authors:** Changwei Song, Shibo Xu, Linna Chang, Xingjun Zhao, Xifan Mei, Xiuli Ren, Zhenhua Chen

**Affiliations:** 1 First Affiliated Hospital of Jinzhou Medical University, Jinzhou 121001, China; 2 Jinzhou Medical University, Jinzhou, Liaoning 121001, China

**Keywords:** cartilage repair, arthritis, tea polyphenol, extracellular vesicles

## Abstract

Arthritis is a kind of chronic inflammatory autoimmune disease, which can destroy joint cartilage and bone, leading to joint pain, joint swelling, and limited mobility. Traditional therapies have many side effects or focus too much on anti-inflammation while neglecting joint repair. In this experiment, we combined Epigallocatechin gallate (EGCG) with extracellular vesicles derived from macrophages to treat rheumatoid arthritis. Sustained-release resulted in a significant decrease in chondrocyte expression of hypoxia-inducible factor 1-alpha, a decrease in apoptosis-related proteins Cytochrome C, Caspase-3, Caspase-9, and Bax. Molecular biological analysis showed that extracellular vesicles-encapsulated EGCG (EVs-EGCG) more significantly upregulated type II collagen expression by about 1.8-fold than EGCG alone, which was more beneficial for arthritis repair. Animal experiments revealed that these EGCG-coated extracellular vesicles significantly reduced swelling, decreased synovial hyperplasia, repaired cartilage, and attenuated arthritis-related pathology scores in arthritic rats. Measurement data showed that EVs-EGCG treatment reduced joint swelling by approximately 39.5% in rheumatoid rats. *In vitro* studies have shown that this EVs-EGCG can increase the expression of cartilage type II collagen and reduce apoptosis of chondrocytes. Moreover, it was demonstrated *in vivo* experiments to reduce cartilage destruction in rheumatoid arthritis rats, providing a solution for the treatment of rheumatoid arthritis.

## Introduction

Arthritis is a prevalent kind of chronic joint disease associated with age that is increasing in prevalence with the aging of the population [1]. Several studies have shown that rheumatoid arthritis is a degenerative joint disease caused by various factors, mainly chronic progressive cartilage degeneration, but can also involve synovium, meniscus, and other factors non-inflammatory disease of multiple tissues [2]. Articular cartilage is an integral part of the joint, playing a series of essential functions such as support and dispersion of charge load. Its structural integrity is significantly damaged in rheumatoid arthritis. The structural integrity of the articular cartilage, which is a significant part of the joint and performs several vital functions such as supporting and distributing electrical load, suffers significant disruption in arthritis [3]. In the cartilage tissue, chondrocytes are the only cell component and have the important function of secreting cartilage matrix and cartilage phenotype. The primary collagen of cartilage is collagen type II, which functions to maintain the architecture and morphology of cartilage. The development of rheumatoid arthritis leads to a decrease in Collagen type II expression. Hence, the study of the role played by collagen type II in rheumatoid arthritis is of great significance for the treatment of arthritis [4, 5]. Moreover, as apoptosis of chondrocytes hinders the body’s cartilage repair, it gradually degrades the cartilage extracellular matrix and eventually leads to loss of joint function [6]. For these reasons, there is a need to quickly find more effective approaches to solve the above problem in the arthritis field.

Epigallocatechin gallate (EGCG) has antioxidant, antibacterial, and immune-enhancing functions and is becoming the attention of many researchers [7]. Moreover, EGCG has a promising application in the food and pharmaceutical fields. Studies have also shown that EGCG has the effect of promoting wound repair [8], and our study focuses on how EGCG promotes cartilage damage repair. The present treatments are confined to taking some intra-articular steroid injection or non-steroidal anti-inflammatory drugs and analgesics to relieve arthritis symptoms. Such treatments, however, can cause damage to the gastrointestinal and cardiovascular systems if administered over a long period [9]. Although research has suggested that EGCG has latent protective influences against chondrocytes cultured *in vitro*, the ease of oxidation, poor lipid solubility, and decreased activity in the alkaline solutions restrict the administration way and application of EGCG to a great extent [10, 11]. Biomaterials have been investigated in the field of tissue repair [12, 13]. Extracellular vesicles are bilayer lipid vesicles of cellular origin mainly used for intercellular information exchange and material transfer. The similarity to cell membranes gives extracellular vesicles the ability to load hydrophobic and hydrophilic drugs. And due to its excellent properties, such as widespread presence in the organism and low immunogenicity, the extracellular vesicle is an ideal drug carrier [14]. At the same time, the use of exosomes can re-induce the body’s tolerance to its antigens and prevent the onset and progression of the disease. Modified exosomes have the potential to induce macrophage polarization in inflamed tissues to treat osteoarthritis, and synthetic exosome products effectively accumulate in inflamed arthritis to induce a series of anti-inflammatory events through macrophage phenotypic regulation [15, 16]. Compared to other studies, our treatment of rheumatoid arthritis with extracellular vesicle-loaded EGCG inhibited synovial production and promoted cartilage regeneration [15, 17].

**Figure 1.**
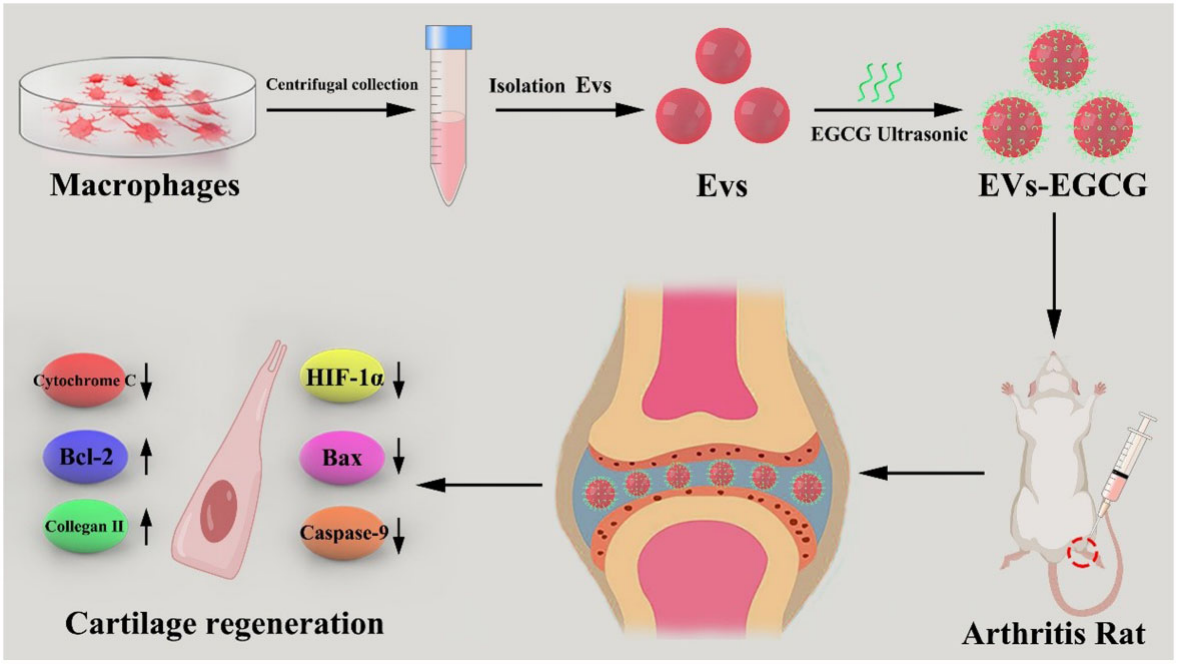
Schematic representation of the assessment of EVs-EGCG regulation of cartilage repair.

Hypoxia-inducible factor 1-alpha (HIF-1α) is a kind of transcription factor that facilitates angiogenesis and exacerbates synovial proliferation. Increased expression of HIF-1α has a regulatory effect on mitosis and apoptosis [18]. It has been shown that mitosis induces chondrocyte degeneration, suggesting that HIF-1α is related to rheumatoid arthritis development [19]. In the present study, we combined extracellular vesicles with EGCG (EVs-EGCG) and used them *in vivo* and *in vitro* experiments while improving the solubility and bioavailability of EGCG. It was found that EGCG encapsulated in extracellular vesicles effectively functioned to reduce chondrocyte apoptosis and increase chondrocyte type II collagen expression in addition to reducing HIF-1α expression. *In vivo* experiments showed that EVs-EGCG significantly reduced joint swelling and the formation of vascular opacification. The experimental results suggest that we have established a new method of extracellular vesicles-encapsulated EGCG for arthritis treatment (Fig. 1).

## Materials and Methods

### Cell culture

Macrophages (RAW), chondrocytes were cultured in DMEM medium (Gibco) and F12 medium (Gibco) involving 10% of fetal bovine serum (FBS; Gibco) along with 1% of penicillin-streptomycin (Gibco). Incubation was performed under the temperature of 37°C (95% humidity, 5% CO_2_), and fluid changes were performed for 2–3 days. When the cell density reached about 80% of the capacity of the culture dish, the cells were digested by utilizing 0.25% of trypsin-EDTA (Gibco) and then passaged.

### Extraction of extracellular vesicles

The supernatant of cultured RAW cells was collected by ultracentrifugation, and dead cells were removed by centrifugation at 15 000 rpm for half an hour and filtered through a membrane. The supernatant after filtration was then centrifuged at 57 000 rpm for 1 h. The morphology and size distribution of the extracellular vesicles were observed by scanning electron microscopy, and the extracellular vesicles were stored at −80°C after precipitation at the bottom. The obtained extracellular vesicles were mixed with EGCG, sonicated in an ice bath, and then suspended, and the process was repeated several times to obtain EVs-EGCG. The sample’s appearance characteristics were displayed through a transmission electron microscope (TEM, JEM-1200EX, Tokyo, Japan). The Zeta potential of the sample was tested by Zeta sizer Nano ZS90 (Thermo Fisher, USA).

### Drug release *in vitro*

EVs-EGCG release assay *in vitro* was studied by phosphate buffer (pH 7.4) as the release medium. Forty milligrams of EGCG and EVs-EGCG were dissolved into the buffer solution (1 ml, with a pH of 7.4), and then the solution was put into a dialysis bag (Spectra/Por Float-A-Lyzer G2, USA). Dip dialysis bag into a centrifuge tube containing 50 ml of the corresponding buffer solution, put them into a water bath constant temperature oscillator, set the oscillation speed to 100 rpm for *in vitro* release test, 4 ml of the solution was taken out of the centrifuge tube at regular intervals to conduct the analysis, and simultaneously, the fresh buffer with identical volume was added into the system.

### Cellular uptake *in vitro*

The obtained extracellular vesicles were labeled in red using the Dil fluorescent labeling kit (Sigma, St. Louis, MO, USA). After that, chondrocytes were incubated with the extracellular vesicles labeled by Dil for three and six hours at 37°C temperature, respectively. Next, the chondrocytes were fixed in 4% of the paraformaldehyde for 30 minutes. Afterward, chondrocytes were treated with 4′,6-diamino-2-phenylindole (DAPI) along with Tubulin (CST, USA). Leica laser scanning confocal microscopy was applied to acquire the images (CLSM, Leica TSCSP5 confocal unit).

### Western blotting analysis

After TNF-α, EGCG, and EVs-EGCG were added to the different cell culture dishes for a period of time. The cells were harvested, cleaned by using PBS, added with the proper amount of the lysis buffer involving FBS, and finally shaken in 100 rpm ice bath for 15 minutes. Then, the solution was centrifuged (15 000 rpm) for 15 minutes at 4°C. BCA Protein Assay Kit was employed to analyze the concentration protein (Pierce, IL, USA) after harvesting supernatant. Each lane with equal amounts was loaded, and SDS-PAGE electrophoresis was utilized to separate the samples, which were subsequently transferred from the SDS-PAGE gels to the membranes of PVDF. They were blocked through utilizing 5% of milk for two hours at environmental temperature and then treated with anti-HIF-1, anti-COLII, anti-Caspase3, anti-Caspase9, anti-Bax, anti-Bcl-2, anti-GAPDH, and anti-Cytochrome C primary antibodies overnight with the temperature of 4°C. Afterward, the samples were treated via the relevant secondary antibodies for 120 minutes. The imaging system of Bio-Rad (Bio-Rad, USA) was applied to display the protein bands, and band intensities were analyzed using ImageJ software.

### Immunofluorescence staining

Each group of chondrocytes was incubated and then washed 3 times with phosphate buffer. After that, the cells were fixed in 4% of the paraformaldehyde for half an hour. Subsequently, the above cells were cleaned three times by PBS, and they were then blocked using 5% of goat serum for two hours. Then, the anti-Col II antibody and HIF-1α antibody were added to the culture medium and stained at 4°C overnight. The cells were subsequently rinsed three times via PBS. And then, they were incubated through the proper secondary antibodies for 120 minutes and cleaned three times using PBS. In the end, these cells were stained with DAPI for 15 minutes. Images were taken by fluorescence microscopy.

### Wistar rat model of collagen-induced arthritis

Male Wistar rats, 4 weeks old, were provided via Changchun Changsheng Biotechnology Co., Ltd. (Changchun, China). All the researches of the animal were accomplished following the Guidelines for Care and Use of Laboratory Animals of Jinzhou Medical University. The rat model of collagen-induced arthritis (CIA) was constructed by utilizing Complete Freund’s Adjuvant and bovine type II collagen. Bovine type II collagen 5 mg, dissolved in 2.5 ml of 0.01 M pH 3.2 glacial acetic acid solution, was prepared one day before the experiment, refrigerated at 4°C overnight, and repeatedly emulsified with complete Freund’s adjuvant (2.5 ml) to prepare an emulsion involving bovine type II collagen (1 mg/ml). After the rats were anesthetized, 100 μl of the emulsified bovine type II collagen glacial acetic acid solution was injected subcutaneously into the right hind toe pad of the rats, and the injection was reinforced again 21 days later to establish the collagen-induced arthritis model.

### Therapeutic efficacy study

The rats were divided into 3 groups as PBS, EGCG, and EVs-EGCG. The treatment was carried out on the 30th day after the initial immunization, and distinct preparations were injected intravenously ten times every other day. The arthritis score and thickness of the hind paw were collected every other day. A clinical arthritis score of 0–4 was assigned to each limb using a semi-quantitative scoring system. Footpad thickness was measured using a micrometer. On day 50, rats were executed, and joint tissue was obtained for histological examination.

### Histological analysis

After finishing the treatment, the rats were killed, and the ankle joints of the rats were harvested and fixed with 4% of paraformaldehyde overnight. The ankle joint was decalcified by using 10% EDTA solution for forty days. Embedding the samples in paraffin and then these samples were cut into 4 μm thick sections. The hematoxylin-eosin and Safranin O fast green (SO-FG) staining were utilized to stain tissue sections. Through utilizing a light microscope, the images of the sections could be acquired (Leica DM4000B, Germany). Application of HSS (histopathology score of synovial) to evaluate the ankle synovial histological changes along with cartilage erosion.

### Statistical analysis

All quantitative data were expressed as the mean ± SD of at a minimum of three measurements. Univariate analysis of variance was employed to test the significance between the groups. The significance was determined with the below thresholds: *P < 0.05, **P < 0.01, ***P < 0.001.

## Results and discussion

### Material characterization

**Figure 2.**
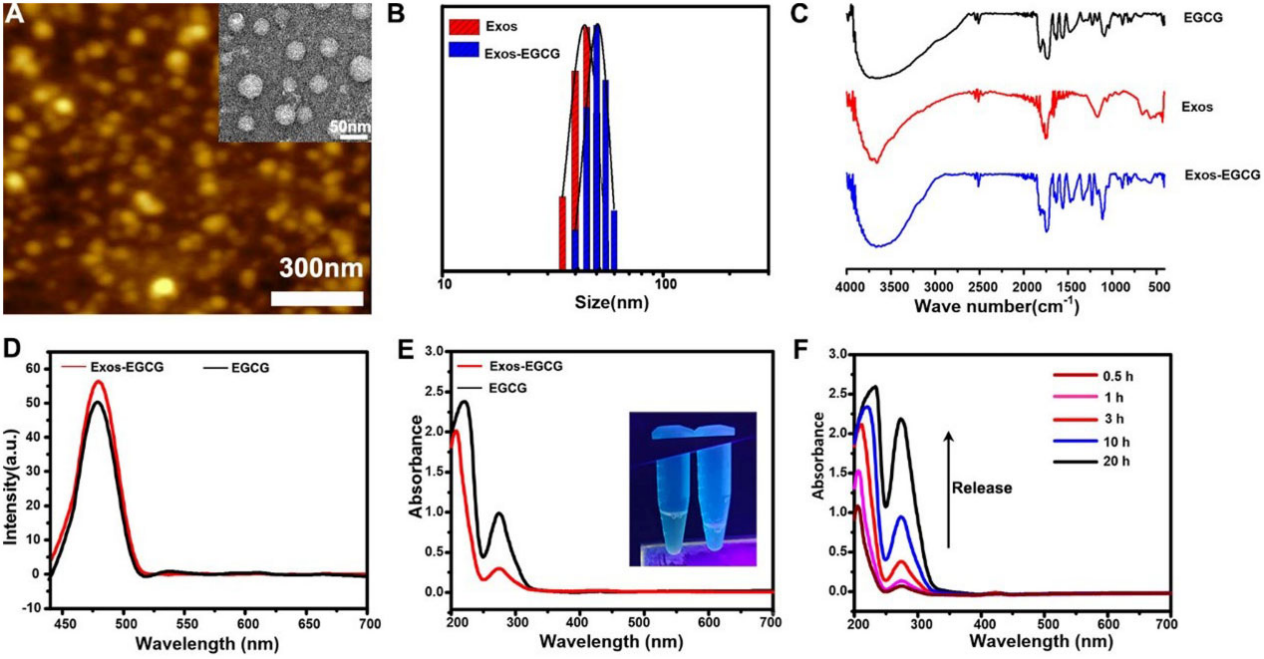
(**A**) AFM image of the extracellular vesicles, the inset in Fig. A was TEM image; (**B**) the DLS size distribution; FTIR spectra (**C**); steady-state fluorescence spectra (**D**) and UV-vis (**E**) of EVs-EGCG, the inset was a photograph of EVs-EGCG (1) and EVs (2) under 365 nm ultraviolet light irradiation; (**F**) UV absorption of EVs-EGCG at different release times.

DLS and AFM were applied to characterize the created products (Fig. 2A and B). The outcomes indicated that the particle size of the prepared extracellular vesicles was about 45 nm, and the prepared EVs-EGCG stock solution had favorable morphology and dispersion with an average particle size of about 50 nm. This might be due to the loading of EGCG, which may change the hydration radius of the nanoparticles. The zeta potential showed a potential of −13.14 mV for EVs and −18.8 mV for EVs-EGCG (Supplementary Fig. S1A). Western blotting results showed that the extracellular vesicles marker proteins CD9, CD63, and TSG101 were expressed in EVs and EVs-EGCG (Supplementary Fig. S1B). It is well known that the composition of extracellular vesicles is similar to cell membranes. The successful synthesis of EVs-EGCG was verified by the stretching vibrational peak of P=O at 1167 cm^−1^ from extracellular vesicles and the feature peak for the benzene ring backbone between 1650 and 1530 cm^−1^ in tea polyphenols (Fig. 2C). The presence of a distinct emission peak of EVs-EGCG at 479 nm (λ_ex_ = 412 nm) was verified by fluorescence spectroscopy (Fig. 2D). As shown in Fig. 2E, EVs-EGCG did not show a clear UV absorption peak, while EVs-EGCG revealed a clear ultraviolet absorption peak at 273 and 220 nm.

Meanwhile, EVs-EGCG showed an apparent green fluorescence under UV light irradiation at 364 nm, while extracellular vesicles showed no color. We also tested the release of EGCG from EVs-EGCG by UV-Vis spectrophotometry and dialysis bags. Figure 2F showed that the amount of EGCG released from EVs-EGCG increased with increasing time. This was due to the presence of extracellular vesicles where EGCG was released slowly and smoothly.

### *In vitro* cellular uptake

**Figure 3.**
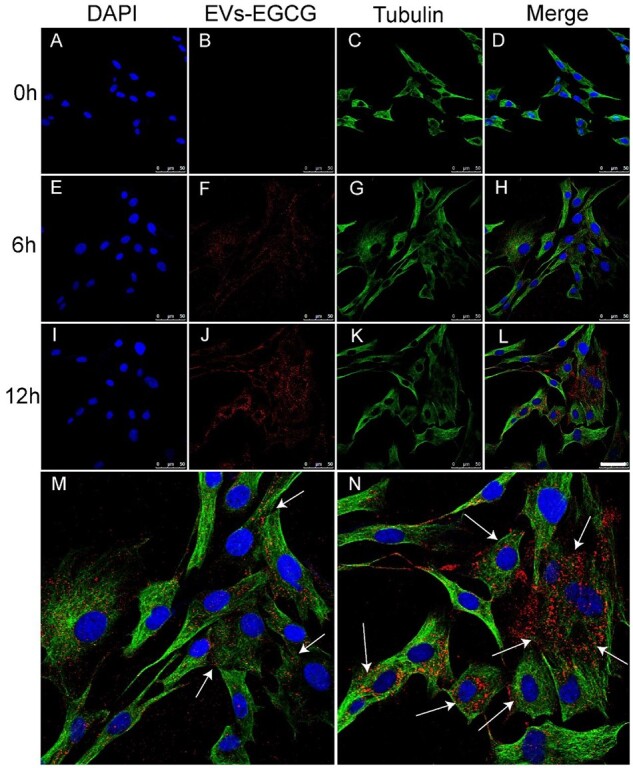
Confocal photography of chondrocyte uptake of extracellular vesicles at 0, 3 and 6 h, respectively, bar = 50 μm (**A**–**L**). (**M**) and (**N**) were the local enlargements of (H) and (L), respectively.

The prerequisite for EVs-EGCG to function is based on the effective uptake of the drug itself by chondrocytes. For this purpose, macrophage-derived extracellular vesicles were labeled with Dil, and when the cell density reached 70–80%, the labeled extracellular vesicles were incubated with chondrocytes. The uptake of extracellular vesicles by chondrocytes was observed by confocal microscopy after 0, 6 and 12 h of incubation (Fig. 3A–L). To better show the uptake, partial enlargements are presented at the bottom left and bottom right for 6 and 12 h, respectively (Fig. 3M and N).

The outcomes suggested that the extracellular vesicles labeled by Dil were slowly migrated to chondrocytes over time, and they were principally around the chondrocyte nucleus. We also found that the uptake of extracellular vesicles by chondrocytes was significantly greater at 12 h than at 6 h with increasing time. These phenomena support our speculation that chondrocytes have a higher uptake rate of Dil-labeled extracellular vesicles.

### EVs-EGCG decreased the expression of HIF-1α

**Figure 4.**
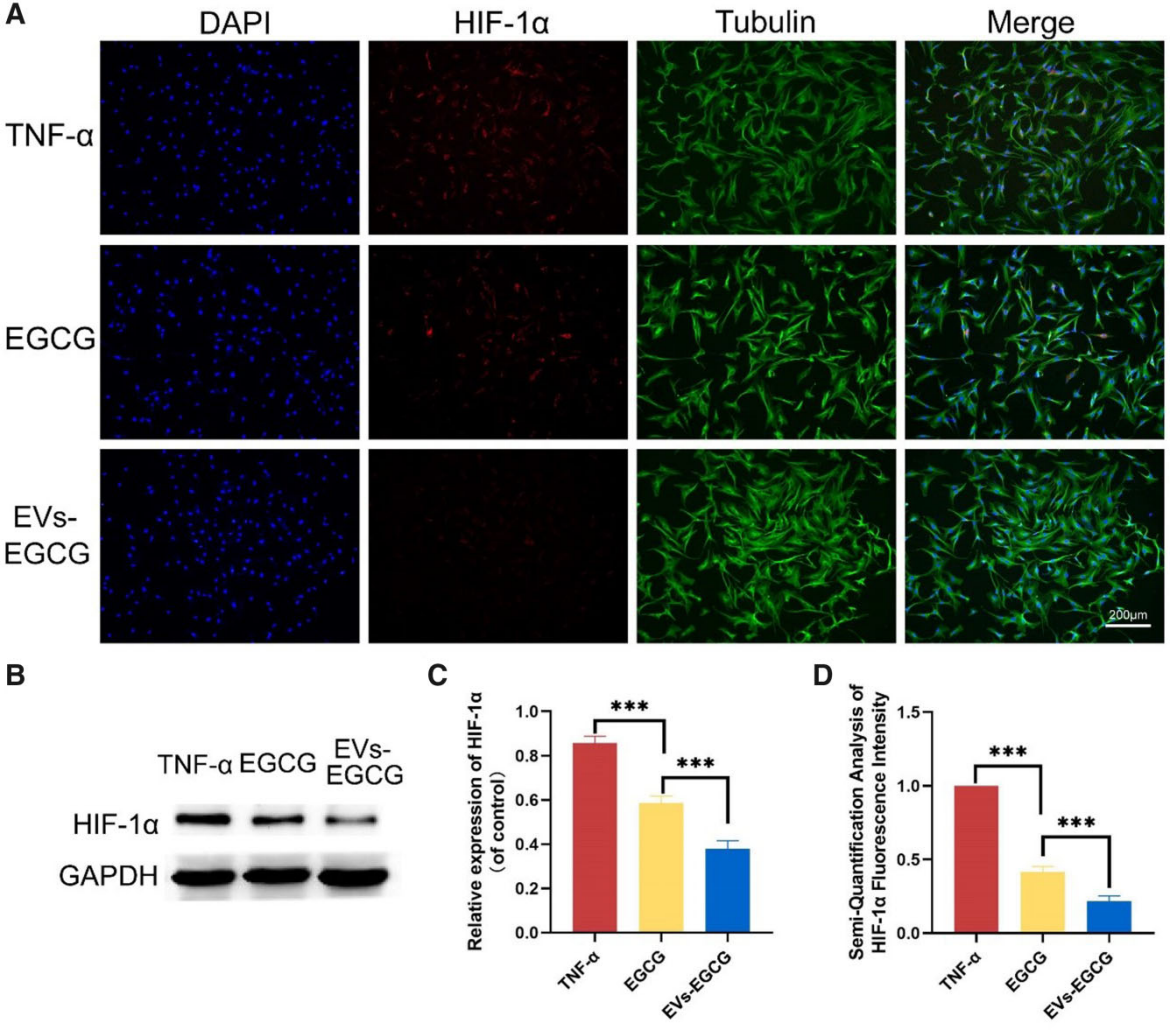
Immunofluorescence and the Western blot to determine the HIF-1α expression in the chondrocytes. (**A**) Fluorescence microscopy of HIF-1α expression in chondrocytes, bar = 200 μm. HIF-1α expression in the chondrocytes was detected (**B**) and quantified (**C**) by Western blot. (**D**) Semiquantitative analysis of fluorescence intensity for expression of HIF-1α in the chondrocytes. *P < 0.05, **P < 0.01, ***P < 0.001.

Cartilage degeneration is a prolonged and complex process that begins with surface cracks, followed by damage that extends into deeper layers, eventually leading to significant loss of cartilage tissue and continuing as the lesion involves the subchondral bone and other tissues of the joint. Because the only cellular component in cartilage is the chondrocyte, the investigation of chondrocytes *in vitro* is important. Cartilage is an avascular tissue with restricted oxygen supply and diffusion, and mitochondria in cartilage play a role in adaptation to hypoxia in a hypoxic environment [20]. In response to changes in the inflammatory microenvironment in arthritis, the HIF is activated and overexpressed to regulate angiogenesis and other processes associated with inflammation [15, 21]. Moreover, HIF-1α is a kind of transcription factor, which facilitates angiogenesis, promotes the formation of vascular opacities, and exacerbates synovial proliferation. Significant expression of HIF-1α could be observed after 24 h induction of rat chondrocytes with TNF-α (Fig. 4A and D). And with the treatment of EGCG or EVs-EGCG, it could be seen that the HIF-1α expression was obviously decreased. In contrast, the HIF-1 expression in EGCG or EVs-EGCG-treated chondrocytes was significantly decreased, with the EVs-EGCG group expressing lower amounts than the other groups.

The application of EVs-EGCG led to an evident reduction in the HIF-1α expression, as demonstrated by western blot experiments and quantitative analysis (Fig. 4B and C). These experimental results suggest that the application of EVs-EGCG may benefit chondrocyte repair.

### Protective effect of EVs-EGCG on chondrocytes *in vitro*

Articular cartilage principally consists of extracellular matrix and chondrocytes. In arthritis, there is an imbalance in the regulation of the anabolic and catabolic activities of chondrocytes, which allows degradation of the chondrocyte matrix. In contrast, the extracellular matrix of cartilage is principally constructed from protein polymorphic aggregates and type II collagen [22].

**Figure 5.**
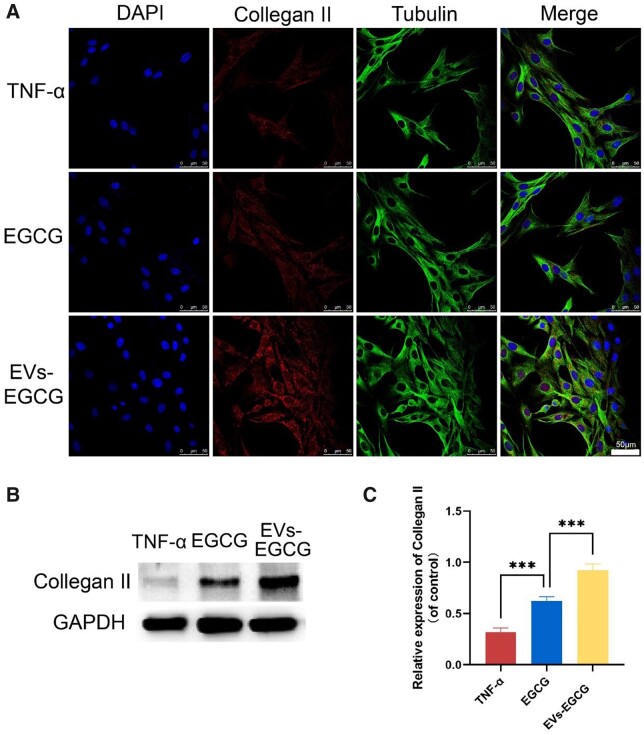
*In vitro* protective influence of EVs-EGCG on chondrocytes under inflammatory conditions. (**A**) Confocal microscopy of expression of type II collagen in the chondrocytes, which were treated via TNF-α and co-cultured with EGCG or EVs-EGCG, bar = 200 μm. Type II collagen expression in the chondrocytes was detected (**B**) and quantified (**C**) through Western blot. *P < 0.05, **P < 0.01, ***P < 0.001.

The articular cartilage’s reticular skeletal architecture principally consists of type II collagen, making the cartilage flexible and resistant to compression [23]. We then used fluorescence confocal to examine type II collagen expression in chondrocytes as a method to assess the repair status of chondrocytes (Fig. 5A). We simulated the inflammatory microenvironment during the pathogenesis of rheumatoid arthritis with TNF-α. Immunofluorescence showed that chondrocytes treated with EVs-EGCG expressed type II collagen greater than the TNF-α-treated group alone. Through western blot and relative western blot quantitative analysis, it was shown that the addition of EGCG effectively increased type II collagen expression. At the same time, the treatment with EVs-EGCG led to a further up-regulation in type II collagen expression (Fig. 5B and C). It should be noted that it may be due to a delay in the rate at which EGCG is oxidized.

### EVs-EGCG exerts a protective effect by attenuating chondrocyte apoptosis

Furthermore, arthritis is an age-related rheumatic disease, which is characterized by chondrocyte apoptosis and articular cartilage degeneration [24]. To further explore the mechanism of the role of EVs-EGCG in arthritis, we further validated the role of EVs-EGCG in arthritis as an inhibitor of chondrocyte apoptosis, based on the demonstration that EVs-EGCG decreased the HIF-1α expression and promoted the production of type II collagen.

**Figure 6.**
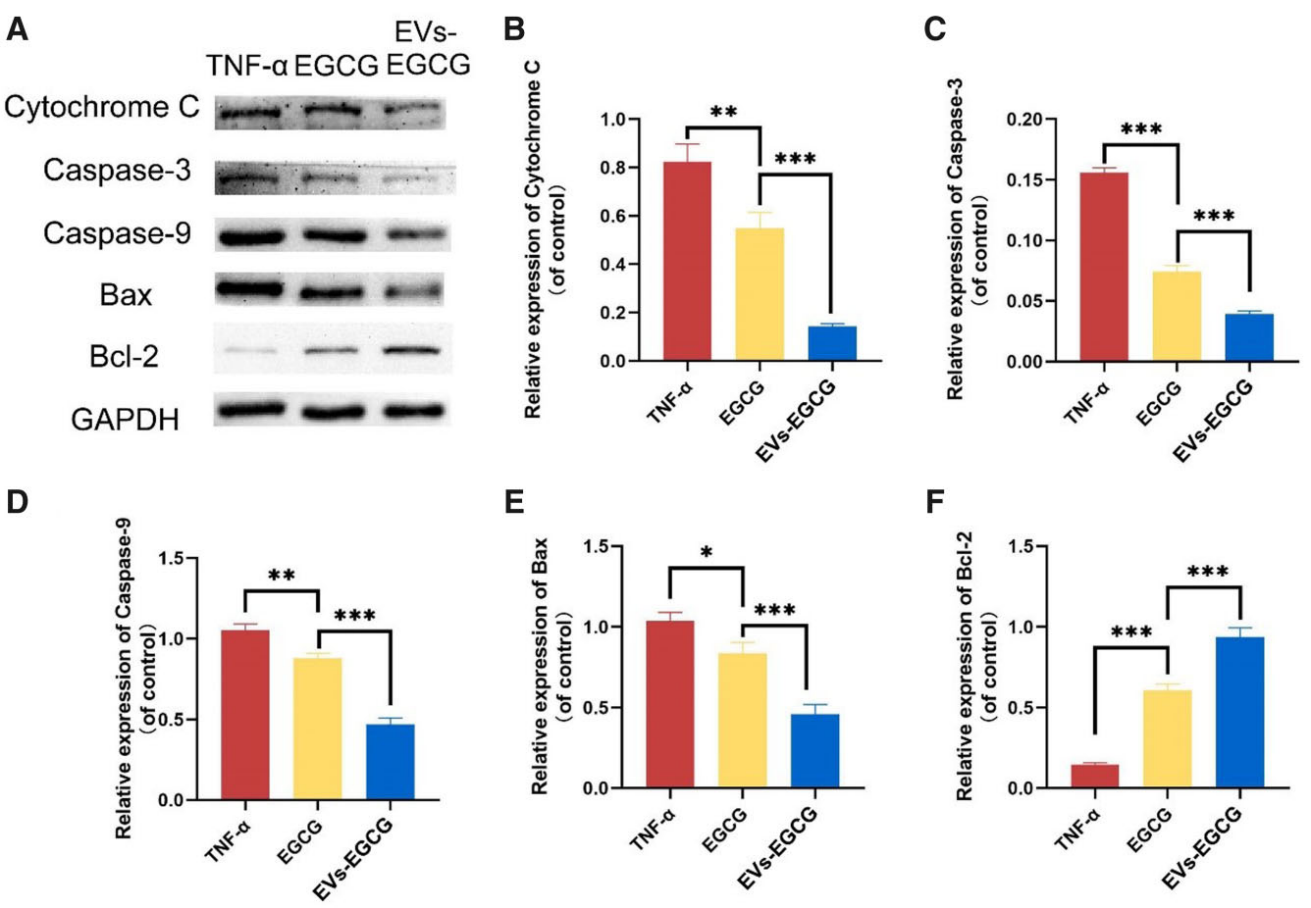
Changes in apoptosis-related protein expression in chondrocytes after EVs-EGCG treatment. (**A**) Expression of apoptosis-associated proteins in chondrocytes by Western blot. The relative expression analysis of Cytochrome C (**B**), Caspase-3 (**C**), Caspase-9 (**D**), Bax (**E**) and Bcl-2 (**F**) in chondrocytes. *P < 0.05, **P < 0.01, ***P < 0.001.

We measured the indexes related to chondrocyte apoptosis in the TNF-α group, EGCG group, and EVs-EGCG group by Western blot, respectively (Fig. 6A). The expression of apoptosis-related proteins decreased to different degrees in all experimental groups, among which the increased Cytochrome C expression level can be regarded as a marker of mitochondrial damage, which leads to apoptosis of chondrocytes due to the increase of Cytochrome C in the course of rheumatoid arthritis disease. We found that the expression of Cytochrome C decreased in both the EGCG group and EVs-EGCG group (Fig. 6B), while the expression of pro-apoptotic proteins Caspase-3 (Fig. 6C), Caspase-9 (Fig. 6D), and Bax (Fig. 6E) were remarkably lower in the group of EVs-EGCG, in contrast to that of the other experimental groups, while anti-apoptotic protein Bcl-2 expression level (Fig. 6F) was evidently increased. The above results showed that we could conclude that both the EGCG group and EVs-EGCG group showed some degree of inhibition of apoptotic proteins, thus proving the repairing effect of EVs-EGCG on chondrocytes in rheumatoid arthritis.

### EVs-EGCG improves arthritis symptoms in rats *in vivo*

**Figure 7.**
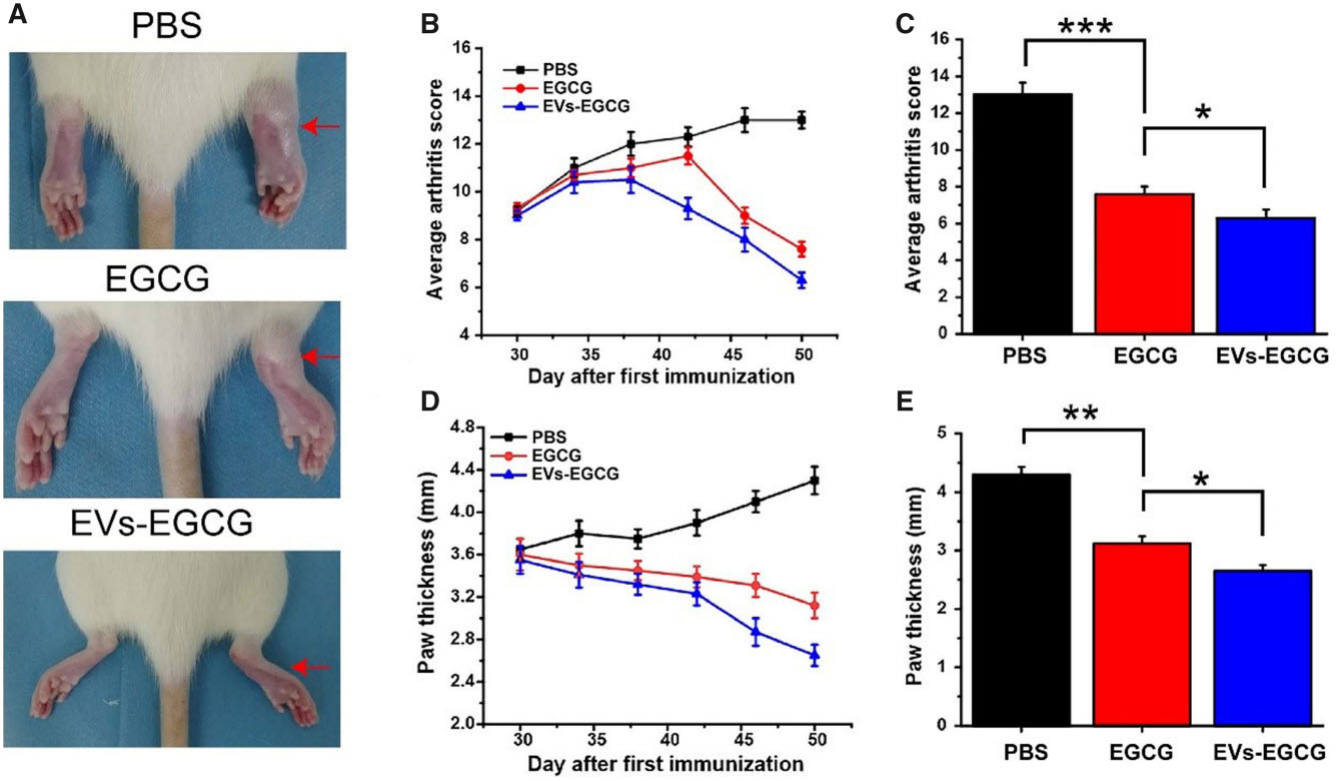
Therapeutic effect of EVs-EGCG on CIA rats. (**A**) Pictures of the inflammatory joints after conducting various treatments. (**B**) Clinical scores of different treatments for arthritis based on time. (**C**) Clinical scores following 20 days with different therapy. (**D**) Claw thickness of inflamed joints with different treatment methods at different times. (**E**) The thickness of claw after twenty days of diverse treatment options.

Finally, the therapeutic effect of EVs-EGCG treatment was evaluated in CIA rats. After 30 days of induced arthritis, CIA rats showed severe swelling of the ankles and paws. Rats with successful CIA modeling were treated with EVs-EGCG for 20 days, during which time the degree of joint swelling was observed and photographed for comparison at the corresponding times (Fig. 7A). To demonstrate therapeutic effect, it was also compared with PBS or EGCG intra-articular injections. The results revealed that both EGCG alone and EVs-EGCG intra-articular injection treatment resulted in significant improvements in the rat joints’ degree of swelling and redness (Fig. 7B and C). Although EGCG showed some therapeutic effect, there is no doubt that EVs-EGCG achieved more significantly lower clinical scores and better final results. In addition to this, the swelling rate was quantified by measuring the thickness of the swollen joints, and for each group, the outcomes were by the scoring results (Fig. 7D and E).

**Figure 8.**
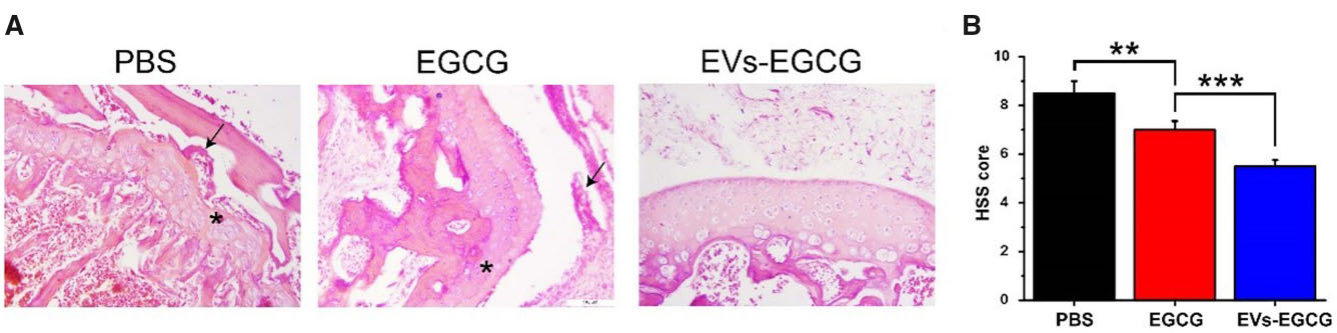
The sections and scores of cartilage tissue in different treatment groups. (**A**) Hematoxylin-eosin staining of rats’ joints in distinct treatment groups. Arrows represent the generation of vascular opacities, and asterisks indicate cartilage destruction. (**B**) Pathological evaluation of synovial tissue determined by HSS.

**Figure 9.**
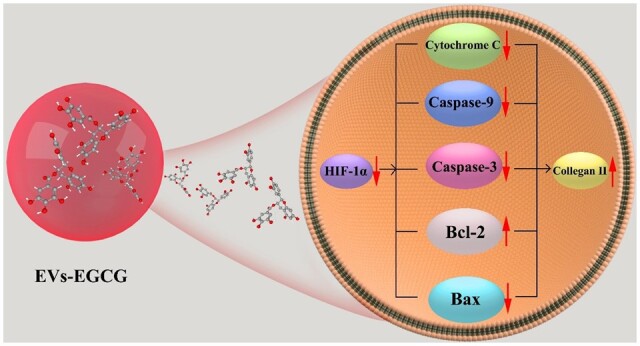
Mechanisms by which EVs-EGCG reduces HIF-1α expression decrease chondrocyte apoptosis and direct cartilage repair.

After that, we further verified our previous judgments by histological experiments. CIA rats treated with PBS alone exhibited marked inflammation with lesions accumulating throughout the synovial, subchondral bone, and cartilage tissues, with cartilage destruction, the proliferation of synovial membrane, and formation of vascular opacities being particularly evident. EGCG-treated and EVs-EGCG-treated groups showed significant improvement in symptoms, reduction in synovial cells, and reduction in cartilage destruction. Both treatment groups showed better architecture than control group. Among them, EVs-EGCG -treated mice showed the mildest inflammation and smooth cartilage surface (Fig. 8A). In addition to this, we quantified histological changes of arthritic cartilage erosion and synovial inflammation. The EVs-EGCG group showed a lower score of histological changes (Fig. 8B). Compared to previous studies, this is a more promising treatment [25]. In addition, SO-FG staining showed smoother joint surfaces in the treated ankles (Supplementary Fig. S2). In addition, immunohistochemistry showed strong expression of apoptotic proteins in the PBS group. In contrast, the expression of apoptotic proteins was weaker in the EGCG and EVs-EGCG groups, and the cartilage surface was smoother in the EVs-EGCG group (Supplementary Fig. S3). The results suggested that EVs-EGCG has an anti-apoptotic effect. Western experiments between the ankle joints of each group further showed the above results. Apoptotic protein expression was significantly lower in EGCG and EVs-EGCG compared to the PBS group. The results indicated that EGCG and EVs-EGCG have significant anti-apoptotic effects, with EVs-EGCG having the best anti-apoptotic effect (Supplementary Fig. S4). Taken together, all these *in vivo* experimental results suggested that EVs-EGCG has a better therapeutic effect compared to EGCG. In conclusion, extracellular vesicular materials show considerable potential compared to other targeted therapies [26]. To better describe the role played by EVs-EGCG, Fig. 9 summarized the mechanism regarding the anti-apoptotic promotion of cartilage repair by EVs-EGCG.

## Conclusion

In conclusion, we created EVs-EGCG using extracellular vesicles and EGCG, which reveals a remarkable inhibitory influence against the development of rheumatoid arthritis disease. Our study showed that EVs-EGCG could down-regulate the expression of HIF-1α, inhibit apoptosis of chondrocytes, and promote the recovery of type II collagen. Animal experiments demonstrated that EVs-EGCG significantly reduced swelling, decreased synovial proliferation, repaired cartilage, and attenuated arthritis-related pathological scores in arthritic rats. Measurement data showed that EVs-EGCG treatment reduced joint swelling in rheumatoid rats by approximately 39.5%, providing a solution for treating rheumatoid arthritis. In conclusion, exosome materials show considerable potential to be developed as the next generation of RA drugs.

## Supplementary data

Supplementary data are available at *REGBIO* online.

## Supplementary Material

rbab067_Supplementary_Data
