# Supplementary material for: Preparation of EGCG decorated, injectable extracellular vesicles for cartilage repair in rat arthritis
Source: Regen Biomater. 2021 Nov 18;8(6):rbab067. doi: 10.1093/rb/rbab067 (PMC8634449; doi:10.1093/rb/rbab067)
Supplement: rbab067_Supplementary_Data [file rbab067_supplementary_data.docx]

**Supporting Information**

**
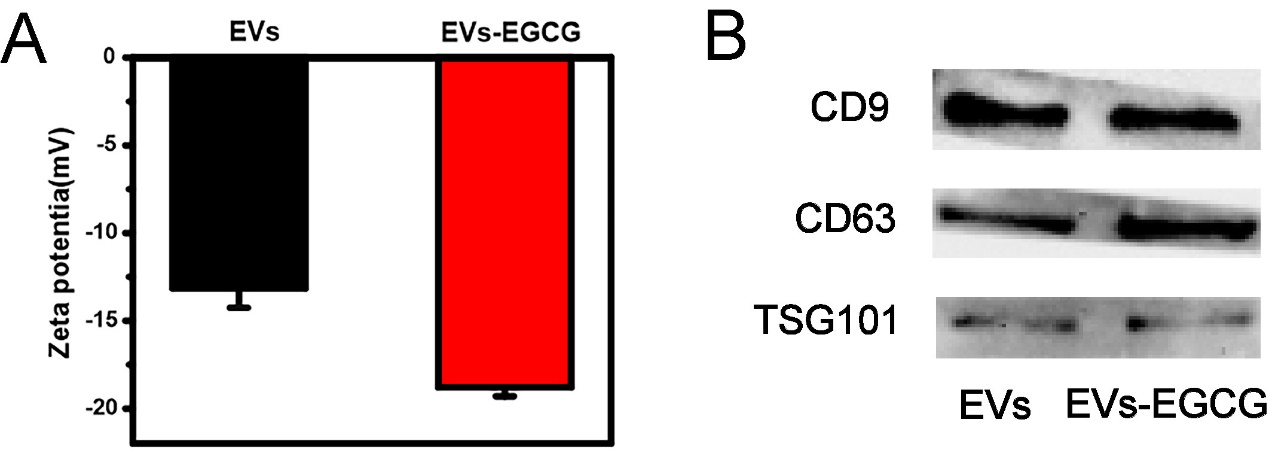
**

**Fig. S1.** (A) The particle Zeta potential distribution of EVs and EVs-EGCG; (B) Western blotting analysis of protein markers of EVs and EVs-EGCG.

**
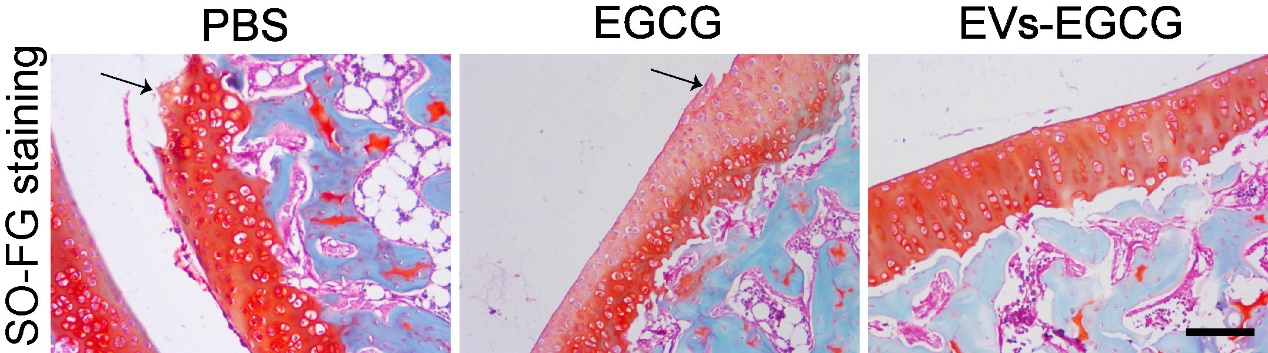
**

**Fig. S2.** Safarnin O fast green (SO-FG) staining of rats’ joints in distinct treatment groups. Arrows represent cartilage destruction, bar=100μm.

**
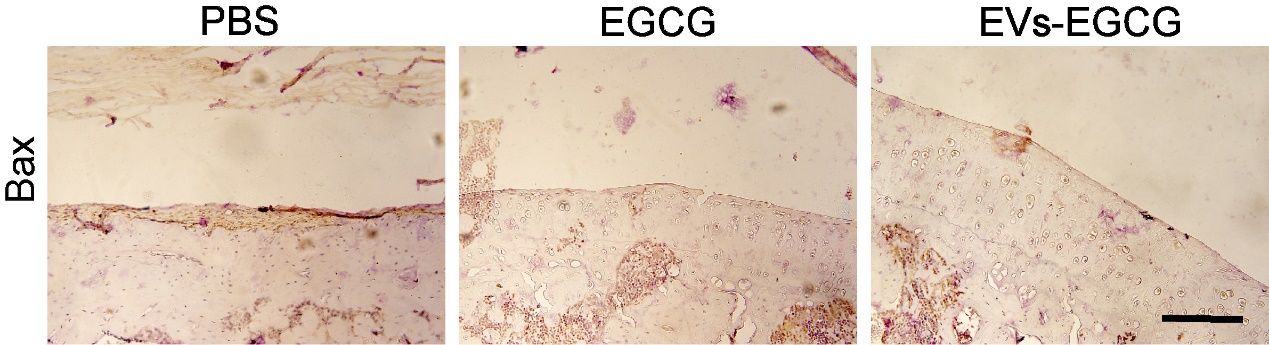
 Fig. S3.** Immunohistochemical staining images of cartilage tissue from different treatment groups, bar=100μm.


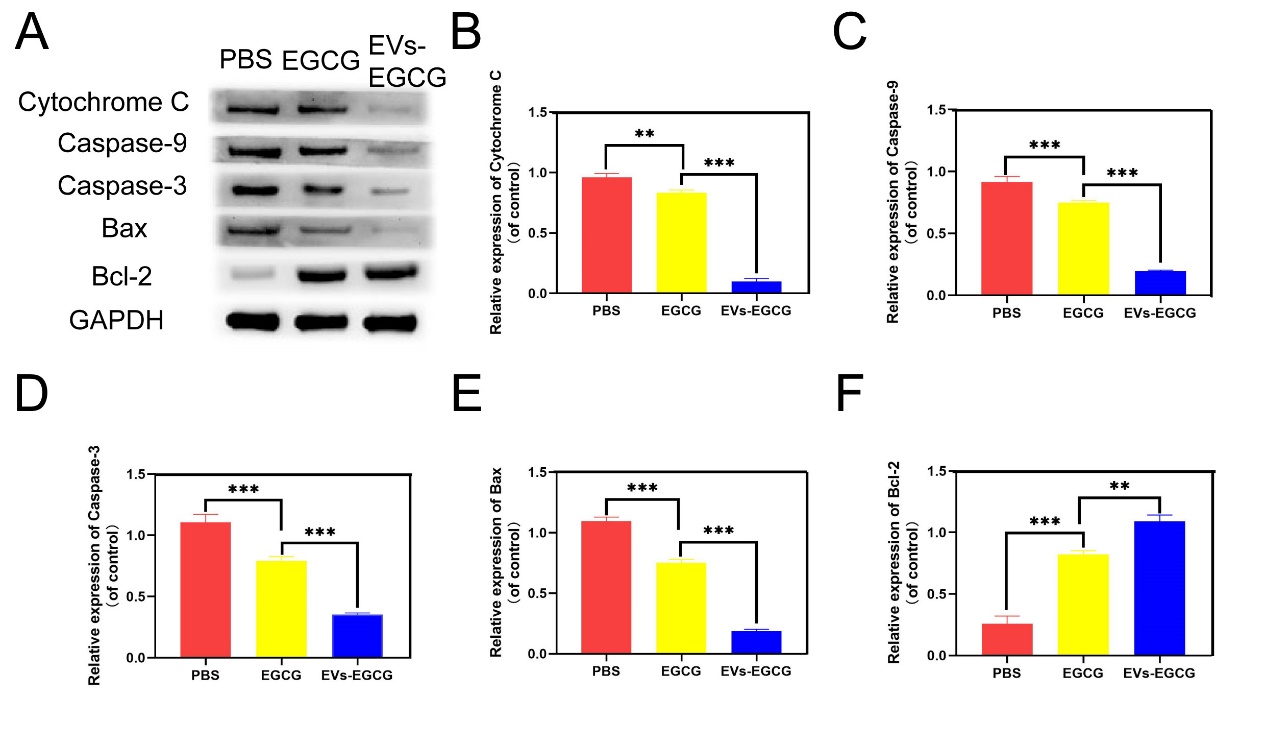


**Fig. S4.** Changes in apoptosis-related protein expression in the ankle joint after EVs-EGCG treatment. (A) Expression of apoptosis-associated proteins in the ankle joint was detected by Western blot. The relative expression analysis of Cytochrome C (B), Caspase-9 (C), Caspase-3 (D), Bax (E), and Bcl-2 (F) in chondrocytes. *P < 0.05, **P < 0.01, ***P < 0.001.
